# Effects of the selection process on malondialdehyde, catalase, superoxide dismutase levels, and the performance of gilts under tropical environmental conditions

**DOI:** 10.14202/vetworld.2023.526-535

**Published:** 2023-03-21

**Authors:** Prester Chuka John Okafor, Nitipong Homwong

**Affiliations:** 1 Department of Animal Science, Faculty of Agriculture at Kamphaeng Saen, Kasetsart University, Kamphaeng Saen Campus, Nakhon Pathom, 73140, Thailand; 2 National Swine Research and Training Center, Faculty of Agriculture at Kamphaeng Saen, Kasetsart University, Kamphaeng Saen Campus, Nakhon Pathom, 73140, Thailand

**Keywords:** antioxidant, gilt, oxidative, stress, tropical

## Abstract

**Background and Aim:**

Gilt selection has the propensity to improve reproductive performance and promote longevity. However, the impact of this process on oxidative stress biomarker levels remains to be unraveled under tropical conditions. This study aimed to determine the effect of management processes during gilt selection on serum malondialdehyde (MDA), catalase (CAT), superoxide dismutase (SOD) levels, and the performance of replacement gilts under tropical environmental conditions.

**Materials and Methods:**

Two groups of 90 crossbred gilts (mean age: 9.72 ± 0.097 weeks) were selected 2 weeks apart, allotted to six pens of 30, and raised in an open barn with shaded roofs. Following their respective entry weeks, gilts in groups one and two were subjected separately to three subsequent selection processes (involving movements, handling, and examination of structural and reproductive traits) at weeks 12, 17, and 24 in the replacement barn. Grower, finisher, and gestation diets were supplied *ad libitum* for 25 weeks. Environmental temperature (°C), humidity (%), and light (lux) were recorded. Malondialdehyde, CAT, and SOD levels were assayed using blood samples collected on day 1 of experiment (PRE), and at week 24 in replacement barn (POST).

**Results:**

Feed intake, weight gain, and percent selected at week 24 were 1.89 versus 1.90 kg/day, 0.81 versus 0.76 kg/day, and 75.23% versus 64.45% for groups one and two, respectively. Sickness, death, slow growth, leg, and reproductive problems caused 24.76% versus 35.55% of removals in groups one and two, respectively. Serum biomarkers were insignificant at PRE but were elevated at POST, with mean values of 14.25 versus 13.84 uM, 5.10 versus 3.26 nmol/min/mL, and p < 0.05, and 1.13 versus 1.68 U/mL and p < 0.05 for MDA, CAT, and SOD in groups one and two, respectively.

**Conclusion:**

The impact of the selection process was meager compared to the adverse effect of high environmental temperatures. The management and selection of replacement gilts in an uncontrolled environmental temperature increase the risk of oxidative stress, especially in tropical regions.

## Introduction

During gilt development, the term “gilt selection” refers to scrutinization processes for retaining phenotypic traits of high reproductive potential within a herd [1, 2]. As sows are expected to farrow 2.4 litters yearly, their feet and legs must be sturdy and large, with evenly sized toes to accommodate the weight increase over time. The pastern should be relatively curved and neither erect nor rigid. The front knee and rear hock angle should support the body cavity such that extreme pressure is not exerted on the leg joints. The ideal foot should also be sternly rigid against the floor without rotating or twisting during locomotion. Splay foot, sickled hock, cow hock, buck knee, and pigeon toes are undesirable traits for reproduction [3]. The vulva and mammary glands should be adequately developed for mating and milking. Vulva must be adequately sized and neither infantile nor tipped up. An average of seven prominent, evenly spaced breast teats devoid of the pin and inverted nipples are desirable [4]. Proper gilt selection can improve reproductive performance and promote longevity [5]. However, ineffective stress management can mar the selection process [6].

Studies have shown that gilts are sensitive to extreme temperatures, high stocking density, starvation, limited access to water, mechanical injury, or aggressive handling [7]. Above 28°C, it has been observed that feed intake and reproductive performance can be impaired in gilts [8, 9]. When challenged with stressors, liver damage, vulva, mammary gland inflammation, rectal prolapse, and reduced nutrient metabolism have also been observed in gilts [10]. The effect of oxidative stress often culminates in lengthy exposure to stressors, leading to the release of various biomarkers [11]. Three biomarkers, malondialdehyde (MDA), catalase (CAT), and superoxide dismutase (SOD), play a crucial role as frontline defense mechanisms in a state of oxidative stress. The stability of these biomarkers, ease of detection, and rapid response have led to their extensive role in studying oxidative stress [12].

Understanding the effect of gilt selection on the level of these biomarkers can help producers adopt best management practices to alleviate the impact of stressors, improving the welfare of developing gilts and overall production efficiency. In tropical countries, such as Thailand, the impact of the selection process could be exacerbated under high temperatures, leading to elevated biomarker activity. Managing the impact of environmental temperature demands a total drift toward intensive farming systems. However, extensive and semi-intensive systems are still common in tropical regions, contributing substantially to overall production output [13].

Therefore, this study aimed to determine the effects of management processes during selection on serum MDA, CAT, SOD levels, and the performance of replacement gilts under tropical environmental conditions.

## Materials and Methods

### Ethical approval

The study was approved by Institutional Animal Care and Use Committee of Kasetsart University, Thailand (ACKU62-AGK-006).

### Study period and location

The study was conducted from March to September 2019. Rearing of gilts and data collection were carried out at a commercial farm located in the western region of Thailand.

### Experimental design

In a randomized complete block design, 180 crossbred nursery gilts (50% Landrace × 50% Yorkshire), with an average of 9.72 ± 0.10 weeks of age and 29.5 ± 1.7 kg body mass, were blocked by selection week in two groups of 90 gilts and moved to an open replacement barn with shaded rooftop. The stocking density was limited to 30 gilts per pen (dimension=4.7 × 8.5 m^2^, equivalent to 1 pig/1.33 m^2^) to reduce stress, with each group having three pens. Following their respective entry weeks, gilts in groups one and two were subjected separately to three subsequent selection processes (involving movements, handling, and examination of structural and reproductive traits) at weeks 12, 17, and 24 in the replacement barn (see Gilt selection below). The average ages of the gilts during these periods were 22, 27, and 34 weeks respectively. Gilts received standard grower (metabolizable energy [ME] = 3,300 kcal/kg and 18.5% crude protein, weeks 1–5), finisher (ME = 3,230 kcal/kg and 17.37% crude protein, weeks 6–18), and gestation diets (ME = 2,950 kcal/kg and 14.00% crude protein, weeks 19–25) during this period. In addition, chelated organic copper (Cu), zinc (Zn), and manganese (Mn) were supplemented at dietary inclusion rates of 0.18, 0.054, and 0.18 g/kg of feed, respectively. All diets were formulated to meet the nutrient requirements for developing gilts [14] and supplied *ad libitum* for 25 weeks. Barn temperature (°C), humidity (%), and light (lux) were recorded thrice daily (9:00 am, 2:00 pm, and 5:00 pm) from March 23 to September 28 using an environmental meter (Extech^®^ EN300, Taiwan).

### Gilt selection

Health conditions, structural, and reproductive traits were examined as a standard process during development. The scrutinization process was conducted in a selection booth. The curvature of the fore and hind leg pasterns, claw length and size, front- and rear-view stance, number and conformation of breast teat, body weight, and locomotion were examined during the first selection. At week 17, the breast teat conformation, vulva length, vulva conformation, gait, and body weight were examined. All structural and reproductive traits were re-examined at week 24 during the third selection. Back fat was also measured from the rump at the position of the last rib using a caliper.

A body condition score cart containing visual descriptions of all anatomical landmarks was used as a guide during the examination. Each body condition was assigned an ordinal score, except for body weight, back fat, vulva length, and the number of breast teats. Each trait was assigned a categorical rank score according to deviations from an ideal anatomical conformation. Gilts having difficulty in locomotion, fewer than 14 functional breast teats, infantile vulva, tipped up vulva, sick, slow growth, or perceived as unfit for reproduction were excluded from the trial [4].

### Blood sample collection

Two milliliters of blood were collected from each gilt on day 1 of the experiment (PRE) and at week 24 (POST) during the last selection by jugular venipuncture. Samples were collected in coagulant serum-collection tubes and centrifuged at 2500× *g* for 15 min at 4°C. The resulting serum was carefully transferred to 1.5 mL microtubes, labeled, and stored at −20°C for further analysis [15].

### Biomarker studies

#### Preparation of samples and standards (MDA assay)

The experimental procedure was adapted with modification [16]. Standards containing 1.25, 2.5, 5, 10, 20, 25, and 50 μM of MDA tetrabutylammonium (99.9%) (Sigma-Aldrich, USA) were prepared from an initial stock of 125 μM. A derivatizing agent (0.1 mL of 10% trichloroacetic acid) (Sigma-Aldrich) was added to 0.1 mL of samples and standards in microtubes, followed by 0.8 mL of color reagent containing 1.06% thiobarbituric acid, 20% glacial acetic acid, and 3.5 M sodium hydroxide. All mixtures were vortexed and heated at 100°C in a water bath for 1 h. Reaction tubes were removed from a water bath, incubated in ice for 10 min, and centrifuged at 1600× *g* for 10 min at 4°C. Aliquots of the resulting supernatants were carefully transferred into microplate wells (in duplicate) for absorbance reading (ABSR).

#### Preparation of samples and standards (CAT assay)

Catalase activity was determined according to the method described [17]. Standard concentrations of 5, 15, 30, 45, 60, and 75 μM were prepared in sample buffer (250 mM potassium phosphate buffer, pH 7.5 containing 0.1% bovine serum albumin, and 1 mM ethylenediamine-tetra acetic acid [EDTA]) using a formaldehyde standard (99.9%, VWR Prolabo®, France). A 0.5095 mg/mL bovine liver CAT (Sigma-Aldrich) was used as a positive control. aliquots of 0.1 mL of assay buffer (100 mM potassium phosphate buffer, pH 7.0) and 0.02 mL of each serum, standard, and positive control were mixed with 0.03 mL of methanol in designated reaction wells in a microplate to perform the assay. The reaction was initiated by adding 0.02 mL of 40 mM hydrogen peroxide and incubating it on an orbital shaker (VRN-360, Gemmy, Taiwan) at 1× *g* for 20 min at room temperature (25°C). A total of 0.03 mL each of 0.01 mM potassium hydroxide and 63 mM of 4-amino-3-hydrazino-5-mercapto-1,2,4-triazole (Sigma-Aldrich) was added to each reaction well and incubated for 10 min. A solution of 0.01 mL of potassium periodate (20 mM) was added to all reaction wells and incubated on an orbital shaker at 1× *g* for 5 min. The microplate was transferred to a spectrophotometer (Multiskan Sky, Thermo Fisher Scientific, USA) for ABSR.

#### Preparation of samples and standards (SOD assay)

The assay was adapted with modifications from [18]. Superoxide dismutase standards and xanthine oxidase were purchased from Sigma-Aldrich. Before assaying, all reagents were equilibrated to 25°C. A stock solution of 0.01 mg/mL SOD standard was diluted to 1.2, 2.4, 4.8, 7.2, 9.6, and 12 U/mL concentrations with sample buffer (1 × potassium phosphate buffer pH 7.8). Aliquots of 0.15 mL assay buffer (0.02 M EDTA, 0.02 M xanthine, and 0.01 M triphenyl tetrazolium chloride (Sigma Aldrich) were first added to all reaction wells in the microplate. Subsequently, 0.02 mL of each sample and standard was added to the designated wells. The enzymatic reaction was initiated by adding 0.02 mL of xanthine oxidase (0.0125 mg/mL) to all reaction wells and incubated at room temperature on an orbital shaker at 1× *g* for 30 min. The microplate was transferred to a spectrophotometer (Multiskan Sky) for ABSR [18].

### Spectrophotometry and calculations

Thirty serum samples were randomly selected for biomarker studies. Samples and standards were prepared as described above. Distilled water, assay, and sample buffers were used as blanks in the MDA, CAT, and SOD assays respectively. All samples collected on day 1 (PRE) were assayed in two replicates. However, for samples collected at week 24 (POST), five replicates were assayed, two within (intra), and three on different (inter) days. Absorbance readings were obtained using a spectrophotometer (Multiskan Sky^®^, Thermo Fisher Scientific, USA) at 530 nm, 540 nm, and 460 nm for MDA, CAT, and SOD, respectively. The calibration temperature was set at 25°C. Absorbance reading was extrapolated into the following equation to estimate serum MDA concentrations:


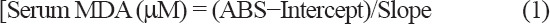


Slope and intercepts were obtained directly from the linear regression equation. Serum formaldehyde concentrations were estimated by extrapolating ABSR values into Equation 2 below. Catalase activity was further calculated using the following equation:


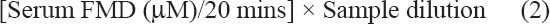


where FMD = formaldehyde. One unit of CAT can be defined as the amount required to release 1 nmol of formaldehyde per minute at 25°C. Catalase activity was expressed as nanomoles per minute per mL (nmol/min/mL) [19].


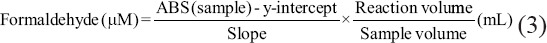



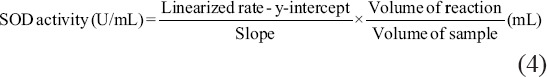


Oxidation of tetrazolium chloride to formazan was inversely related to SOD concentration. Hence, a linearized rate was obtained by dividing blank ABSR by itself and all standards and samples. The sample linearized rate was extrapolated into Equation 4 to estimate SOD activity. One unit of SOD is the amount that can dismutate 50% of superoxide radicals in a biological matrix, and SOD activity was expressed as units per ml of SOD (U/mL) [18].

### Validation and quality assurance

All validation processes were conducted statistically using the spectrophotometric analysis results. Limits of detection (LOD) and limits of quantification (LOQ), intra- and inter-assay precision, and percent recovery rate were determined for all biomarker assays. The LOD and LOQ were calculated from a linear regression equation using the following formula:


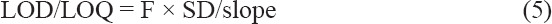


where F is a constant for the signal-to-noise ratio (the acceptable F values for LOD and LOQ are 3.3 and 10, respectively). The terms SD and slope represent the residual standard deviation and slope of the linear regression. Intra- and inter-day assay precisions were determined and expressed as percentage coefficient of variation (%) [20].

### Statistical analysis

Variance homogeneity between groups was analyzed using Bartlett’s test [21]. Group biomarker levels, average daily feed intake (ADFI), average daily weight gain (ADG), feed conversion ratio (FCR), the impact of environmental conditions on ADFI, the effect of temperature, humidity, and light on growth performance, and monthly environmental temperature were analyzed using a general linear model [22]. The percentage of gilts selected, culled, dead, and removal causes were analyzed using a generalized linear model with the probit link function. Trends in environmental temperature, humidity, and light, predicted death probability, and temperature impact on death incidence rate were all modeled using locally weighted regression. All statistical analyses were performed with R version 4.1(www.r-project.org) [23] with the ”ggplot2” package (www.ggplot2.tidyverse.org) [24] for graphical presentation and the “labdsv” package (www.ecology.msu.montana.edu/labdsv/R) [25] for binomial modeling. Statistical significance was set at p < 0.05.

## Results

### Growth performance and gilt selection

**Figure-1:**
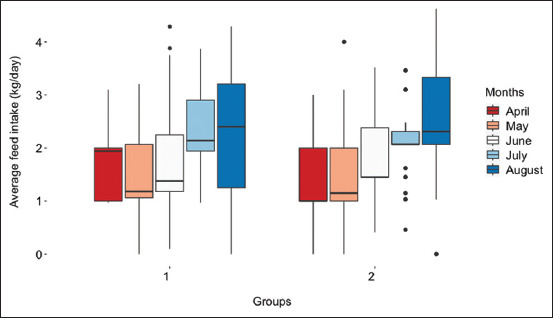
Average monthly feed consumption in two groups of replacements raised in an open barn. Feed consumption increased linearly as months advanced with the lowest record observed in April and the highest in August.

The graph in Figure-1 shows a progressive increase in monthly feed intake with age by group. Growth performance was statistically insignificant across the groups. The estimated averages for ADFI, ADG, and FCR in groups one and two are shown in Table-1. Two major decisions were resolved at each selection stage: to “retain” or “cull.” From week 12, the point of the first decision, to week 24, after the third selection, there was a decline in the percentage of gilts retained and an increase in culled gilts. No death incidents were observed after the first selection. In addition to death, other causes of removal were sickness, leg problems, reproductive problems (slow vulva and mammary gland development), sickness, and slow growth (Table-1).

**Table-1:** Comparing feed efficiency, environmental effect, selection rate, removal rate, and causes of removal for gilts.

Parameters	Mean		SE	p-value

	Group 1	Group 2		
Feed efficiency				
ADFI^1^, kg	1.89	1.90	0.040	0.904
ADG^1^, kg	0.81	0.76	0.020	0.156
FCR^1¶^	2.56	2.49	0.160	0.766
Environmental effect				
Env-ADFI^1^, kg	1.89	1.90	0.050	0.988
Temperature^1^, oC	31.86	31.83	0.150	0.887
Humidity^1^, %	65.74	66.03	0.610	0.748
Light1, lux	387.14	370.41	38.095	0.831
Selected gilts, %				
1^st^ decision^2^	85.12	87.07	6.005	0.862
2^nd^ decision^2^	78.58	87.53	6.430	0.458
3^rd^ decision^2^	75.23	64.45	7.890	0.457
Culled gilts, %				
1^st^ decision^2^	5.33	7.54	4.255	0.782
2^nd^ decision^2^	11.45	6.80	4.980	0.620
3^rd^ decision^2^	15.85	30.36	7.170	0.272
Dead gilts, %				
1^st^ decision^2^	8.51	5.31	4.395	0.689
2^nd^ decision	0.00	0.00	0.000	NE
3^rd^ decision	0.00	0.00	0.000	NE
Removal causes, %				
Dead^2^	8.51	5.31	4.395	0.689
Lame^2^	0.00	6.21	3.350	0.092
Leg problem^2^	2.37	3.12	2.835	0.876
Reproductive problem^2^	2.46	0.99	2.240	0.725
Sickness2	10.19	10.90	5.330	0.944
Slow growth^2^	1.69	9.48	3.850	0.232
Miscellenous^2^	1.05	2.34	2.225	0.757
Total removal^2^	24.76	35.55	7.895	0.457

1 p-values obtained from a general linear model.

2 p-values obtained from a generalized linear model using a binomial distribution with the probit link function. ^¶^Values estimated at pen level as gilts were group-fed. Env-ADFI=Average daily feed intake adjusted for environmental effect, ADG=Average daily weight gain, ADFI=Average daily feed intake, FCR=Feed conversion ratio, NE=Not estimable, SE=Standard error

### Environmental parameters and mortality

The impact of environmental conditions on gilt performance was also examined during the study. There was no statistical difference in the average monthly temperature, humidity, or light in pens across the groups (Table-1). The highest average temperature (33.83°C) occurred in March and the lowest (30.51°C) in August. The sunlight radiation varied monthly, with the highest intensity occurring between March and May. The humidity had an inverse relationship with temperature. The lowest monthly average (55%) occurred in April, and the highest (71%) occurred in June (Table-2).

**Table-2:** Average monthly temperature, light, and humidity in barn during the trial period.

Months	Humidity (%)		Light (lux)		Temperature (°C)	
		
	Mean	SE	Mean	SE	Mean	SE
March	55.51	3.181	532.79	79.640	33.83	1.066
April	57.07	1.207	533.61	88.471	33.77	0.287
May	63.77	1.682	531.56	78.192	32.59	0.343
June	71.63	1.552	205.33	22.287	31.22	0.450
July	68.97	1.424	221.46	26.777	30.83	0.273
August	70.32	1.570	145.6	17.166	30.51	0.281
September	70.74	2.128	85.72	10.049	30.80	0.702

Temperature and light intensity have a monthly decreasing trend; humidity had an opposite trend, increasing progressively as the months advanced.

SE=Standard error

**Figure-2:**
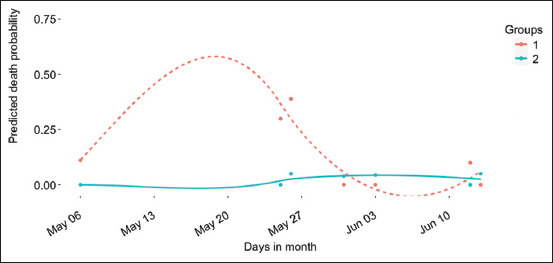
Predicted death probability regressed on days in month. Death incidents were observed in the month of May and June with the highest probabilities (0.10, 0.30, and 0.38) on 6^th^, May 25 and 26, respectively. No further deaths occurred beyond June 13. The dotted and continuous lines represent Group 1 and Group 2, respectively.

**Figure-3:**
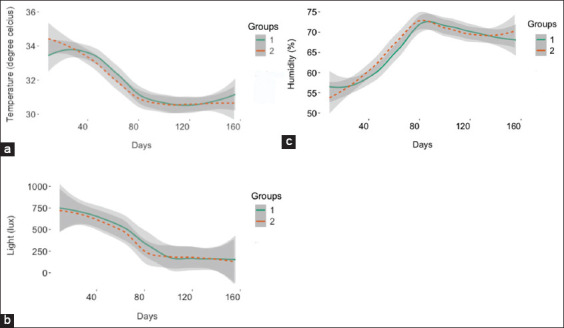
Trend in environmental temperature, humidity, and light for the duration of the gilt development (between March and September, n = 161 days). (a) Temperature (°C) (b) humidity (%) and (c): Sunlight radiation (lux). Graph was obtained from locally weighted regression. Temperature and light had a decreasing trend through the study period while humidity had an increasing trend. Temperature was highest from day 1 to 60, corresponding to days from March 23 and May 21. Average daily temperature declined significantly from day 80 (in June) to approximately 31^o^C through the study period. Humidity increased exponentially, reaching its peak of approximately 74% on day 80 but it never went below 70% thereafter. Light on the other hand decreased from a high of 750 lux on day 1 to values below 250 lux by day 80 and remained so throughout the study. The dotted line represents Group 1 and the continuous, Group 2.

**Figure-4:**
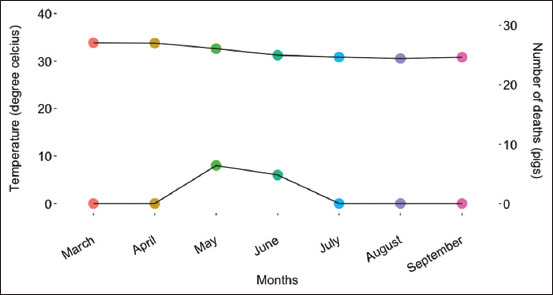
Association between observed death and monthly average temperature. No deaths were observed in March, April, July, and August. In the month of May, a total of 8 death incidents were observed and in June it was 6. The monthly average barn temperatures for these months were 32.59 and 31.22, respectively.

Mortality was a considerable economic and welfare issue during the study. Figure-2 shows the predicted death probability by the date of occurrence. High mortality was observed in group one, although the difference did not have any statistical significance. The highest death incidents were observed between May 25 and 26. The first death occurred in May (average temperature = 32.52°C). This occurred sequel to the lengthy exposure to temperatures above 33°C in March and April. The last incident occurred in June (average temperature = 31.22°C), despite the declining trend in temperature and sunlight. Graphs of daily environmental temperature, humidity, and light are presented in Figures-3a–c. Eight deaths were recorded in May and 6 in June (Figure-4). No subsequent deaths occurred after that. The impact of the selection process and environmental temperature was also estimated by changes in blood biomarker levels.

### Biomarker assay and validation

#### Assays

**Figure-5:**
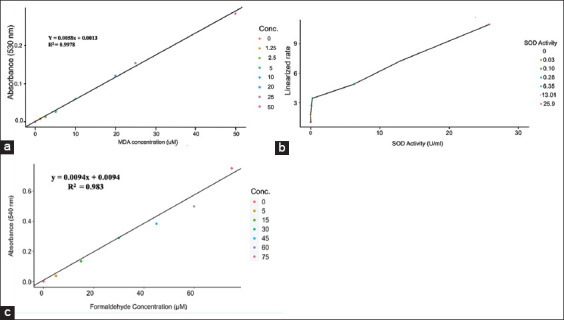
Standard calibration curves for malondialdehyde, superoxide dismutase, and catalase assays obtained by spectrophotometric analysis. (a) The calibration curve for Malondialdehyde (MDA) was obtained by measuring the absorbance of the pink adduct produced by the reaction of MDA with thiobarbituric acid at 100°C. The ABSR linearly corresponds with MDA concentration in sample. (b) Linearized rate of superoxide dismutase (SOD) was calculated from the quotient of blank with itself, standard and sample absorbance. Linearized rate increases with an increase in SOD activity. (c) An indirect peroxidase assay was used to quantify the concentration of formaldehyde released by CAT in the presence of H_2_O_2_.

The ABSR of MDA increased proportionally with the intensity of the pink-colored adduct, producing a calibration curve with a regression coefficient of 0.0058 and a 0.9978 coefficient of determination (Figure-5a). The regression showing the relationship between SOD activity and the linearized rate is shown in Figure-5b. Following the peroxidation pathway, the CAT assay produced a calibration curve with a regression coefficient of 0.0094 and a 0.98 coefficient of determination (Figure-5c).

Serum MDA concentration was statistically insignificant between the groups at PRE and POST. Although the group means were low at PRE. Regarding SOD and CAT, there was a significant increase in POST levels (p < 0.001) (Table-3). At the same time, SOD activity increased by 20.2% and 18.3% in groups one and two, respectively. At POST, there was a corresponding increase of 220.75% and 176.27% in CAT activity in the respective groups. Parameters for assay validation were analyzed for each biomarker and presented in Table-4.

**Table-3:** Comparing malondialdehyde, catalase, and superoxide dismutase levels in blood samples of replacement gilts.

Parameters	Mean		SE	p-value^1^

	Group 1	Group 2		
PRE				
MDA, μM/mL	12.46	11.83	1.178	0.707
SOD, U/mL	0.94	1.42	0.237	0.164
CAT, nmol/min/mL	1.59	1.18	0.409	0.494
POST				
MDA, μM/mL	14.25	13.84	0.954	0.759
SOD, U/mL	1.13^b^	1.68^a^	0.094	< 0.001***
CAT, nmol/min/mL	5.10^a^	3.26^b^	0.354	< 0.001***

1 p-value obtained from a linear model. ^ab^Non-identical superscripts within rows indicate statistically significant means (p < 0.05). *p < 0.05, **p < 0.01,

*** p < 0.001. MDA=Malondialdehyde, CAT=Catalase, SOD=Superoxide dismutase, PRE=Blood samples collected at day 1 of the experiment (10 weeks of age), POST=Blood samples collected at week 24 (34 weeks of age), SE=Standard error

**Table-4:** Parameters for validating spectrophotometric assays of malondialdehyde, catalase, and superoxide dismutase.

Parameters	MDA		CAT		SOD	
		
	Mean	SE	Mean	SE	Mean	SE
LOD	6.28a	2.203	3.5b	1.708	4.02^c^	2.492
LOQ	19.02^a^	6.676	10.82^b^	5.176	12.19^c^	7.553
Recovery (%)	101.25	3.931	106.39	1.832	99.73	3.288
Intra-assay CV (%)	5.99	0.004	9.43	0.048	2.88	0.0004
Inter-assay CV (%)	6.72	0.013	13.62	0.045	2.97	0.001

a MDA concentration (μM/mL).

b AT activity (nmol/min/mL).

c SOD activity (U/mL). LOD=Limit of detection, LOQ=Limit of quantitation, CV=Coefficient of variation, MDA=Malondialdehyde, SOD=Superoxide dismutase, CAT=Catalase, SE=Standard error

## Discussion

This research aimed to determine the effects of management processes during selection on serum MDA, CAT, and SOD levels. The performance of replacement gilts under tropical environmental conditions was also assessed.

### Feed efficiency

There was no significant difference in ADFI between the groups. Quinn *et al*. [26] observed an ADFI of 2.25 kg/day in developing gilts, which is sligltly higher than the 1.9 kg/day in this study. Stalder *et al*. [27] reported an ADFI of 2.1 kg/day for replacement gilts. On the other hand, the ADG values of 0.81 and 0.76 kg observed here were consistent with a previous study [26]. An ADG between 0.6 and 0.8 kg/day has been deemed appropriate for good-performing gilts [28]. Previous studies have shown that ADFI and ADG are affected by feeding mode, management approach, environmental condition, and age [26, 27]. In the current study, although gilts had unrestricted access to feed, the dietary energy level was reduced with each diet phase to harmonize the growth rate and reproductive development. The FCR reported in the current study indicates that approximately 2.5 kg of feed is required for every kg of weight gain. This implies that feed quality is consistent with most commercial variants [26].

### Gilt selection and environmental climate

The percentage of gilts selected and culled at the first, second, and third selection decisions were statistically insignificant across experimental groups. The choice of traits (e.g., wrists, pasterns, front- and rear-view stance, hock conformation, breast teats, and vulva) was based on the anticipated economic value and the likelihood of impacting longevity and future reproductive performance. According to Van Steenbergen [29], five traits responsible for 57% of variability in structural conformation were side view stance of fore and hind leg, locomotion, claw, and frame size. These traits are influenced mainly by changes in body weight. According to Stock *et al.*, [2], other heritable traits likely to influence structural characteristics include pastern and hock conformation. In their suggestion, such traits which are likely to hinder longevity should be evaluated more often. The inconsistency between the current finding and that of Van Steenbergen [29] could be as a result of the evaluation and scoring system. While a linear scoring system was used for evaluating external traits in their study [29], a categorical ranking was applied herein. The deviations of examined features from normal anatomical conformations were ranked using an ordinal score. Although simpler than linear systems, the categorical technique also provides adequate information for genetic inference [30].

It was observed that reproductive problems, difficulty in locomotion, slow growth, and leg problems were the predominant reasons for culling replacement gilts [30]. In contrast, most culls in this study were caused by sickness. Reproductive problems only contributed to 2.46% and 0.99% of total removals in groups one and two, respectively. The high percentage of culled gilts due to sickness must have been a result of acclimatization to the environmental conditions of the barn. This was shown by the increased activity of antioxidants at POST and acute death incidents.

Deaths were recorded only in the first selection during the study. An investigation into the exact causes of these sudden deaths was beyond the scope of this study. However, from statistical inference, there was a strong association between gilt mortality and environmental temperature. The average barn temperature for the duration of the current study was above 30°C, a value deemed detrimental for pigs [31]. Although gilts could adapt under this condition, there was also a reduction in feed intake according to Hörtenhuber *et al*. [32]. It was also shown that gilts can tolerate short-term exposure to environmental temperatures up to 30°C in a controlled environment. However, ambient temperature should optimally be maintained below 28°C in tropical regions [33]. In this study, the effects due to environmental conditions may have been exacerbated because gilts were raised in an open barn. This is typical of tropical regions, especially during the summer seasons (between March and June in Thailand). Environmental conditions can be improved by evaporative cooling systems, which can maintain barn temperatures between 25°C and 27°C [34]. Studies have shown that passive housing and vegetation can also improve ambient conditions [35–37].]. Changes in biomarker level were evaluated to further examine the effects of the selection process on gilt performance.

### Effect of gilt selection on biomarkers

Biomarker levels at PRE were used as control to establish changes during development. The MDA concentrations of 14.25 and 13.84 μM reported herein (for groups one and two, respectively) at POST exceeded the maximum range of 4.45 μM observed by Todorova *et al*. [38]. Ntawubizi *et al*. [15] reported 6.70 μM and 6.26 μM in barrows and finishing pigs, respectively. Silva-Guillen *et al*. [39] observed that feeding gilts with peroxidized lipids for 35 days elevated serum MDA concentration from an initial value of 8.43 μ to 12.50 μ, indicating that prolonged exposure to stressors can have a considerable impact on the MDA concentration. Malondialdehyde values can be influenced by factors such as feed composition, animal species, age, sex, method of analysis, and sample type [38, 40]. Feeds were uniformly formulated, and the same breed, age, and sex were used in the study. The two potential stressors identified in this study were the selection process and seasonal environmental temperature. Apart from fueling gilt mortality, these stressors have also been implicated in increased MDA, CAT, and SOD levels.

Serum SOD activity increased significantly at POST, leading to values of 1.13 U/mL and 1.68 U/mL in groups one and two, respectively. POST levels also correspond with previously reported values for pigs of matched physiological age [41]. The current finding contrasts with the values reported in a study by Vives-Bauza *et al*. [42]. The activity of SOD varies with the type of enzyme (i.e., Mn, Cu, or Zn isoform). For example, it was shown that the Mn and Cu SOD isoforms vary significantly, even when examined in samples from the same animal [42]. While it has been reported that SOD levels in quality semen do not exceed 1.05 U/mL, serum SOD levels can reach up to 200 U/mL in healthy pigs, and in mitochondrial DNA, can exceed 1500 U/mg. This means that SOD can vary by sample type [41, 42]. Other sources of variation are species of animals, the source of stressors that affect each species, physiological age, sex of the animal, and method of assaying [15, 41, 42]. However, it has been shown that the degree of variability differs slightly within species, indicating a conserved transcription mechanism [43]. In pigs challenged with the porcine virus, SOD levels rose by 186.4, 157.6, and 35.7 units in weaners, fatteners, and finishers, respectively, compared to controls [41]. Because sickness was the predominant cause of removal in this study, it can be inferred that increased SOD activity was a biochemical response to an incursive stressor. In a stressed state, the primary structure of SOD might be altered due to oxidative damage. However, the secondary or alpha-helical region is highly conserved and seldom affected by oxidative damage [44]. This explains why there was only a slight increase in SOD activity compared to CAT. The antioxidant defense mechanism was not utterly overwhelmed by the stressor; however, physical evidences presented by number of sicknesses and deaths indicates the degree to which the observed stressors impacted the performance of replacement gilts.

Catalase activity increased and was statistically significant between the groups at POST. The value reported herein was lower than that of Jakimiuk *et al*. [45] for both controls and stress-challenged gilts. Previous studies have shown that CAT activity increases in response to oxidant concentration [44, 46]. In agreement with these observations, the finding in this study indicated that the increase in MDA must have contributed to elevated CAT activity. The type of stressor and the sample being evaluated might contribute to variability when interpreting the test results. Studies have shown that exposure to stressors induces a conformational change in the primary and secondary structure of CAT, allowing the charged metalloenzyme in the catalytic center to bind to free oxygen radicals and hydrogen peroxides. This process neutralizes the effect of oxidants. However, the persistent proliferation of oxidants without countereffects by antioxidants might lead to adverse effects [44]. This process partially explains why sickness and death were the most prevalent causes of gilt removal in the study. Although an essential intervention for improved performance, gilt selection can become distressing under unfavorable environmental conditions.

## Conclusion

To the best knowledge of the authors, this was the first study investigating the effect of gilt selection on oxidant and antioxidant biomarkers under tropical environmental conditions. Growth, feed efficiency, and structural and reproductive development were unaffected by the selection process. Sickness and death significantly affected gilt removal, and more deaths were observed as a result of the hot environmental temperature. Although MDA levels were statistically insignificant between groups, gilt selection significantly impacted SOD and CAT levels, reflecting a biochemical response to a change in redox homeostasis. The effect of the selection process might be considered meager compared to the mortality risk caused by prolonged exposure to uncontrolled environmental temperatures.

## Authors’ Contributions

NH: Conceptualized and designed the study. PCJO and NH: Performed the experiment, analyzed and interpreted the data, and drafted the manuscript. Both authors have read, reviewed, and approved the final manuscript.
